# Complex Network Clustering by a Multi-objective Evolutionary Algorithm Based on Decomposition and Membrane Structure

**DOI:** 10.1038/srep33870

**Published:** 2016-09-27

**Authors:** Ying Ju, Songming Zhang, Ningxiang Ding, Xiangxiang Zeng, Xingyi Zhang

**Affiliations:** 1 School of Information Science and Technology, Xiamen University, Xiamen, China; 2 School of Computer Science and Technology, Anhui University, Anhui, China

## Abstract

The field of complex network clustering is gaining considerable attention in recent years. In this study, a multi-objective evolutionary algorithm based on membranes is proposed to solve the network clustering problem. Population are divided into different membrane structures on average. The evolutionary algorithm is carried out in the membrane structures. The population are eliminated by the vector of membranes. In the proposed method, two evaluation objectives termed as Kernel J-means and Ratio Cut are to be minimized. Extensive experimental studies comparison with state-of-the-art algorithms proves that the proposed algorithm is effective and promising.

Network analysis is an important topic in computer science and bioinformatics researches^1^,^2^,^3^. In the last decade, a large number of algorithms for network clustering, such as the algorithm of Girvan and Newman^4^, fast greedy modularity optimization^5^, and Markov cluster algorithm^6^, were proposed. Much research, such as that by Orman and Labatut^7^ and Forunato^8^, has been conducted on community detection in networks. However, because of the inherent complexity of network clustering, community detection problems often cannot be satisfactorily solved by traditional heuristic optimization methods. Thus, metaheuristic algorithms are adopted as a tool for dealing with community detection problems. These metaheuristic algorithms are notable for their effective local learning and global searching capabilities and have proven to be more successful than heuristic methods in solving optimization problems^9^,^10^,^11^,^12^. Metaheuristic algorithms, which also automatically determine the number of communities, are thus convenient to adopt in community detection applications^13^. Numerous scholars have applied some metaheuristics algorithms, such as evolutionary and particle swarm optimization algorithms, to the problem of network clustering. In this regard, Pizzuti^14^ proposed a single objective genetic algorithm (GA-net) for network clustering; Gong^15^ suggested a memetic algorithm-based network clustering method (Memenet). Although used successfully, in both theory and application in community detection problems, those single objective algorithms still have a significant disadvantage. For example, different algorithms on the same network may produce different solutions. Many single objective algorithms also must determine the number of communities as prior information^16^,^17^. However, for real networks, this information is often unknown.

To alleviate the disadvantages of single objective algorithms, multi-objective optimization is applied to the problem. A large number of multi-objective optimization evolutionary algorithms have been developed, which can be potentially effective and helpful for solving the problem^18^,^19^,^20^. Therefore, using multi-objective optimization algorithms to solve the community detection problem has become a significant subject^21^,^22^. In 2007, Zhang and Li^19^,^20^,^21^ proposed an algorithm, a Multi-Objective Evolutionary Algorithm based on Decomposition MOEA/D). However, research on MOEA/D has revealed that some, but not all, solutions are chosen in several sub-problems (Fig. 1), which may result in loss of population diversity. To address the sub-problems, we designed a new Multi-Objective Evolutionary Algorithm, where each sub-problem has several solutions (Fig. 2).

Membranes play an essential role in the structure and the functioning of living cells. In this study, we propose a novel algorithm, Multi-Objective Evolutionary Algorithm based on Decomposition and Membrane structure (MOEA/DM), for community detection based on an evolutionary algorithm. To solve the problem of a solution corresponding to several sub-problems, we add a membrane structure to help ensure that a sub-problem will have multiple solutions, where the membrane structure refers to the structure of membrane computing models^23^,^24^,^25^,^26^. We seek the optimal solution through the evolution of a particle in the membrane structure and the exchange of an adjacent membrane structure among the optimal particles. Experimental results indicate that, in terms of time and effect, MOEA/DM performs better than MOEA/D.

The rest of this paper is organized as follows. Section 2 describes the community detection and concept of multi-objective optimization. Section 3 elucidates the proposed MOEA/DM. Section 4 presents the experimental studies. Section 5 concludes the study.

## Clustering problem related background

### Network Community detection based on the graph

A network is usually expressed as a graph structure, *G* = *G* (*N, V*), where *N* represents nodes and V represents the relationships between the network’s nodes. For a graph, *G* = *G* (*N, V*) can also be expressed as an adjacent matrix, *A*. For every element *a*_*ij*_ of *A*,
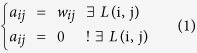
*L*(*i*, *j*) denotes that node i and node j are connected; *w*_*ij*_ represents the weight value of the two nodes.

The purpose of network community detection is to determine the characteristic similarities of the nodes in the network and then classify them. If a network is undirected and unweighted when an edge is connected between two nodes, then *a*_*ij*_ = 1; otherwise, *a*_*ij*_ = 0. The degree of node *i* is defined as 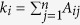. However, the degree of node *i* can also be expressed as 
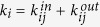
, and expressed as follows:
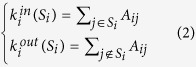


For network we divide into m communities, *S* = {*S*_*1*_, *S*_*2*_, …, *S*_*m*_}, if 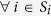, 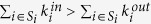, this community is called strong sense, and community in a weak sense if 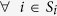, 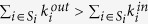. The above statement shows that, in a strong community, each node has more connections within a community than with other communities in the network. In a weak community, the sum of the degrees within the rest of the community, is greater than the sum of the degrees within the community.

### Multi-objective Optimization(MOP)

A multi-objective optimization problem is stated as follows:
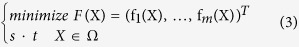
where Ω is the variable space. 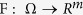 contains m objective functions where *R*^*m*^ is defined as the objective space. Unlike a single objective optimization problem producing one optimal solution, there are probably many, even infinite, solutions for problem (3). These feasible solutions are called Pareto Optimality. Let *u*, *v* ∈ *R*^*m*^. *u* is said to dominate *v* if for any *i* ∈ {1, 2, …, m}, *u*_*i*_ ≥ *v*_*i*_ and there exists at least one *j* ∈ {1, 2, …, m} that *u*_*j*_ > *v*_*j*_. If there is no point *X* ∈ **Ω** such that *X* dominates X^*^, then X^*^ is a Pareto optimal solution. All the non-dominated X^*^ set is called Pareto Front. However, it is time-comsuming and even impossible to find the entire Pareto Front. Therefore, most algorithms aim to find out an even-distributed part of Pareto Front to represent the whole one.

Under certain conditions, a multi-objective optimization problem(MOP) can be decomposed into several single objective optimization problems(SOPs). There are two types of algorithms to decompose an MOP into a group of SOPs. The first type is weight aggregation based decomposition approaches, a set of weight vectors are used to convert an MOP into a number of SOPs using a scalarization method. the weighted Tchebycheff approach^27^ and the PBI approach^21^ are most widely used. The second type decomposes the objective space into a group of subspaces using a set of weight/reference vectors, which are most widely used in recent years.

The Tchebyshev method is classic and is expressed as:
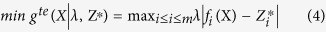
where X ∈ Ω, z* is the reference point. For *i* ∈ [1, m], Z_i_^*^ = *min f*_*i*_ (x). Z_i_^*^ = *min*{ *f*_*i*_ (x)|*x* ∈ Ω}, *i* = {*1*, *2*, …, *m*}. For each non-dominated solution x* of (3), there exists a weight vector lamda so that x* is the optimal solution of (4). We cannot conclude whether or not (1) the pareto front is concave and (2) the two objectives we use in this paper are discontinuous. When the pareto front is non-concave, the weighted sum approach does not work well, and that is why we choose the Tchebycheff approach.

## MOEA/DM for Community Detection

### Introduction of MOEA/DM algorithm

In 2004, MOEA/D was proposed. However, we discovered there is a lack of diversity in the pareto front. We assume the reason for this is there may be a number of sub problems corresponding to the same non-dominated solutions. Therefore, we propose a MOEA/DM algorithm to reduce the number of sub problems and improve the probability that the solution is not the same for each sub-problem and corresponding optimal.

### Objective function

For an unsigned network, the degree of the node reveals the closeness between the nodes. Modularity density (D)^28^, widely used in a variety of community detection algorithms, is one of the most basic measurement standards that uses the degree of the node. D is defined as:
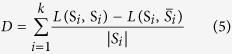
In Eq. (5), each sum means the ratio between the difference of the internal and external degrees of the subgraph *S*_*i*_ and the size of the subgraph. In the above formula, we define 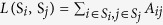 and 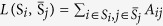. Give a partition *S* = (*S*_*1*_, *S*_*2*_, …, *S*_*m*_) of the graph, where *S*_*i*_ is the vertex set of subgraph *G*_*i*_ (*i* = *1, 2,* …, *m*). However, MOEA/D divided D into two parts as two objectives one of which is NRA (negative ratio association) and the other one is ratio cut (RC).
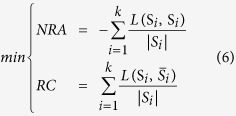


RC is used to measure the connection density between communities and NRA is used to measure connection density within communities. If these two goals are optimized simultaneously These two objectives can be determined to minimize the community more closely and the internal connection between communities sparse connection. Given a partition *S* = (*S*_1_, …, *S*_*m*_), *S*_*i*_ are the decision variables, and *m* is the scale (i.e., the number of decision variables) of the problem.

### Encoding and decoding of discrete population position

**Figure 3:**
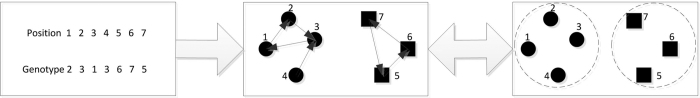
Encode of network.

Proposed in the graph structure is a genetics-based adjacency matrix notation^29^, where each individual, g, of the population consists of *N* genes, each of which, takes allele values, *j*, in the range *1, 2*, …, *N.* Genes and alleles represent nodes of the graph *G* = (*V*, *E*) modeling a network. Thus, *a* value of *j*, assigned to the *i*th gene, is then interpreted as a link between the nodes *i* and *j*, and, in the resulting clustering solution, the nodes are in the same cluster. The decoding of this representation requires the identification of all connected components. All nodes belonging to the same connected component are then assigned to one cluster. A main advantage of this representation is that it is unnecessary to fix the number of clusters in advance, because the number of clusters is automatically determined in the decoding step. Figure 3 illustrates the locus-based adjacency scheme for a network of seven nodes.

### Crossover

**Figure 4:**
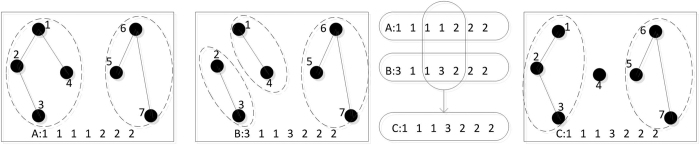
Crossover of solution.

We choose the two-point crossover, in favor of uniform crossover, because the two-point crossover better maintains effective node connections in the network. Given two parents, A and B, we first randomly select two points *i* and *j* (*1* ≤ *i* ≤ *j* ≤ *N*), and then everything between the two points is swapped between the parents (i.e. 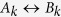,  ∀ *k* ∈ {*k*|*i* ≤ *k* ≤ *j*). An example of the operation of two-point crossover on encoding is shown in Fig. 4.

### Combination of Evolutionary Algorithms and Membrane Structure

The main idea of MOEA/DM is that object space is divided into a plurality of membrane structures and the solution of each membrane structure is initialized. Through population evolution within the membrane to screen out the best solution in each membrane and passed to the adjacent membrane structure. In each evolution of the membrane interior and also remove the worst performance of the solution. So in a sub-problem we choose the best solution is relatively more and to ensure that in each membrane is the best. Through a number of iterations, the solutions of each membrane structure is considered to be the best solutions of the sub-problem that corresponding membrane structure.

**Figure 5:**
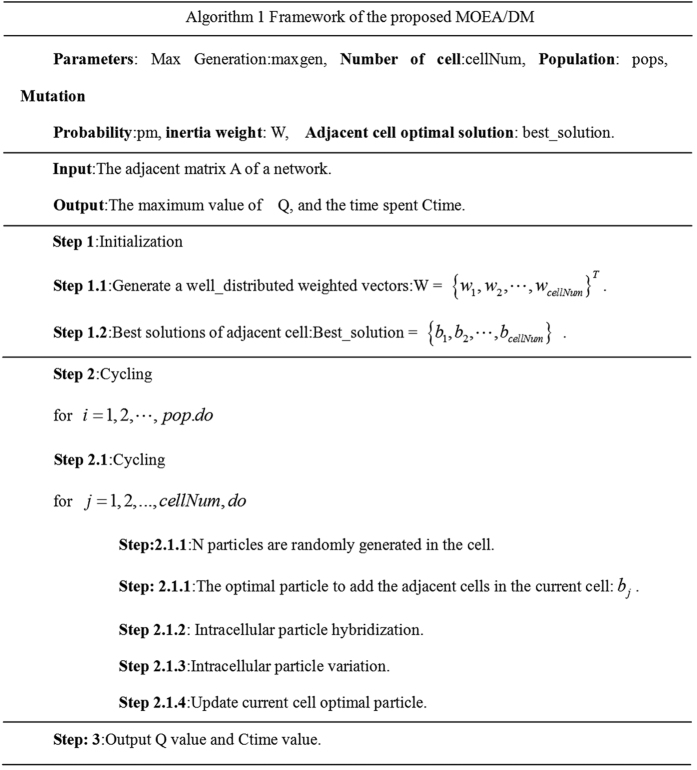
Framework of the proposed MOEA/DM.

Through in comparison to the four known classification network and in two unknown classification network MOEA/D and MOPSO, MOEA/DM in the calculation of the cost of time is much faster than the other two algorithms and also in effect superior by. The algorithm flow can be expressed as Fig. 5.

## Experimental Results

In this paper, we compare, mainly in time and Q^4^,^29^ values, our proposed algorithm with one EA-based algorithm (MOEA/D) and one PSO-based algorithm (MOPSO). Experimental parameters are listed in Table 1. Compare the number of iterations of the three algorithms, those algorithms set for the 200 generation. Finally, we use the modularity proposed by Newman and Girvan. The modularity is defined as:
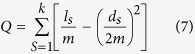


### MOEA/D algorithm

MOEA/D uses the Pareto dominance concept to allow the heuristic to handle problems. The algorithm provides a simple, but efficient, way of introducing decomposition approaches into multi-objective evolutionary computation. A decomposition approach, often developed in the community of mathematical programming, is readily incorporated into EAs in the MOEA/D framework to solve MOPS. Because MOEA/D is better than the EA-based algorithm, we chose MOEA/D for comparison with our proposed algorithm (MOEA/DM) to determine the performance of time cost and Q-value.

### MOPSO algorithm

The MOPSO uses the same Pareto dominance concept to allow the heuristic to handle problems. However, the main difference between MOEA/D and MOPSO is the optimization strategy. Because MOPSO is better than the PSO-based algorithm, we chose MOPSO for comparison with our proposed algorithm (MOEA/DM) to determine the performance of time cost and Q-value.

For each test instance, both MOEA/D and MOPSO were run independently 100 times on the same computer (Inter(R) Celeron(R)M CPU 520 machine, 1.6 GHz, 512 MB memory). The operating system is Windows 8. In our experiments, the following performance indexes are used. There are many parameters that can be set flexibly, shown on Table 1, such as the parameter that are used to store the dominant solution, that we call it niche. The parameter niche is used to determine the neighborhood size and influence on the performance of our algorithm. In order to find the best value, we run the algorithm 200 times with different niche. After a lot of experiments, when niche equals 13 more suitable for this algorithm. Apart from the parameter niche, the population size popsize, the iteration number maxgen and the cell number CellNum are also affected the results of the experiment. We use the method of controlling variables, and ultimately determine their values. We set the popsize equal 120, the gmax equal 200 and the CellNum equal 40.

### Experimental results on real-world networks

In this section, we demonstrate the MOEA/DM application effects on five real-world networks. Of these, the dolphin social^30^, the American college football^31^, the Zachary’s karate club^32^ and the political book network found from V. Krebs are known to be true. For the Santa Fe Institute SFI^33^ and the netscience networks^34^ the true data classification is unknown. The characteristics of the networks are given in Table 2. Table 3, we reflect on the performance of Q and running time on the value of the data that is known to the specific classification of MOEA/DM. Table 4, we reflect on the MOEA/DM performance of the Q value and time cost on the two unknown exact classification data.

### Comparison of algorithms on the karate network

#### Karate network

The Karate network is a social network analysis in the field of classical data sets. In the early 1970s, the sociologist, Zachary, took two years to observe the social relations among the 34 members of an American university karate club. Based on these internal club members as well as external exchanges, he constructed the social relations between members of the network consisting of 34 nodes. An edge between two nodes means that between the corresponding communities at least two members frequent exchanges of friends.

**Figure 6:**
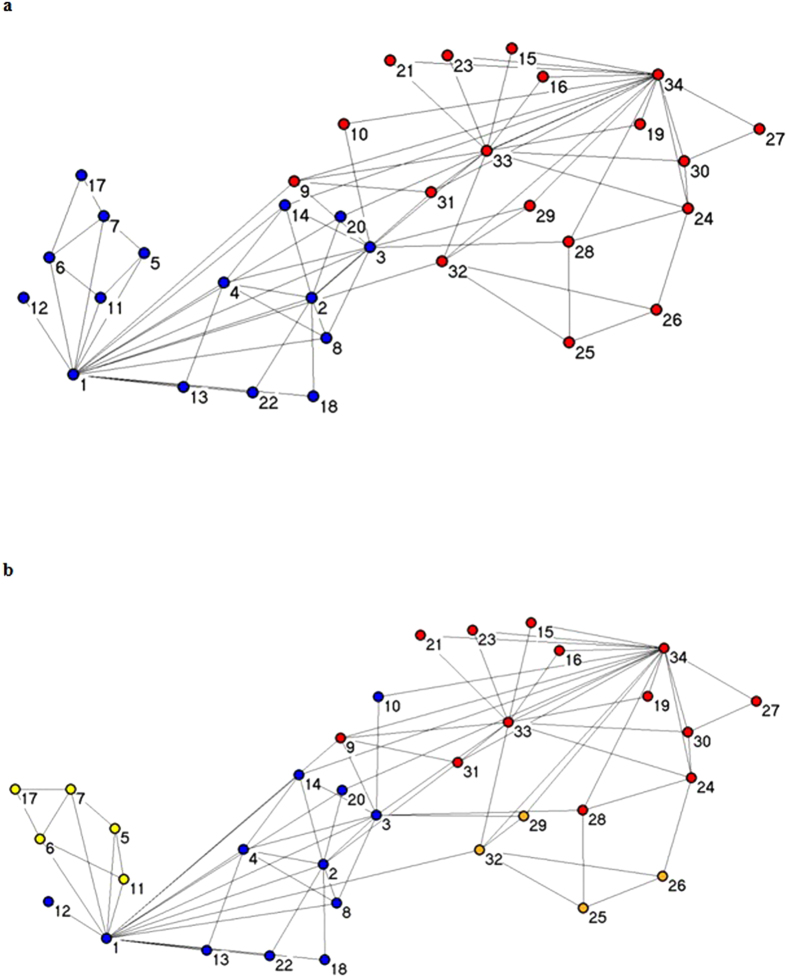
Clustering results on Karate club network by MOEA/DM. (**a**) The real structure of Karate network. (**b**) The prediction structure of Karate network detected by MOEA/DM.

In Fig. 6(a), we show the true situation of the clustering karate network, In Fig. 6(b) we present the results of the clustering algorithm, MOEA/DM. In Fig. 6(b), MOEA/DM is divided into four categories: the top part is divided into two categories and the bottom part also divided into two parts. In Fig. 6(a), Point 10 (red) belongs to the real structure (upper part). According to our prediction, Point 10 (blue) should belong in the predicted structure (lower part) shown in Fig. 6(b). Other papers designate points (such as Point 10) as fussy nodes, i.e., it can be either classified to the first cluster or to the second one.

In the classification process, because points have just two edges to connect two different categories, points (such as Point 10) are divided into two parts. Although we used four categories (rather than two), we correctly divided the network.

Table 3 shows that, although the performance value index, Q, for our MOEA/DM is consistent with the values for MOEA/D and MOPSO, the time value for MOEA/DM is superior to the times for MOEA/D and MOPSO.

### Comparison of algorithms on the dolphin network

#### Dolphin network

**Figure 7:**
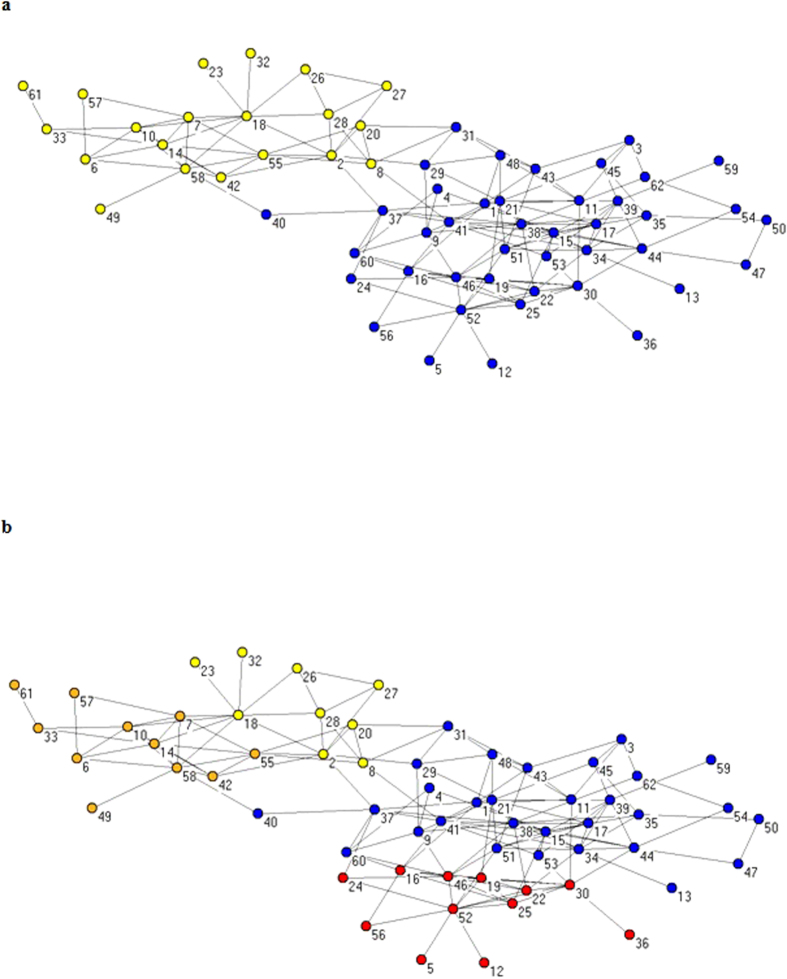
Clustering results on Dolphin club network by MOEA/DM. (**a**) The real structure of Dolphin network. (**b**) The prediction structure of Dolphin club network detected by MOEA/DM.

In New Zealand’s life habits of 62 bottlenose dolphins, Lusseau^30^ found the dolphin’s interaction with a specific pattern, and constructed a social network containing 62 nodes. This dolphin network is naturally separated into two large groups: female and male. In Fig. 7(a), we show the true situation of the clustering dolphin network, In Fig. 7(b) we present the results of the clustering algorithm, MOEA/DM. In Fig. 7(b), MOEA/DM is divided into four categories: the top part is divided into two categories and the bottom part also divided into two parts.

**Figure 8:**
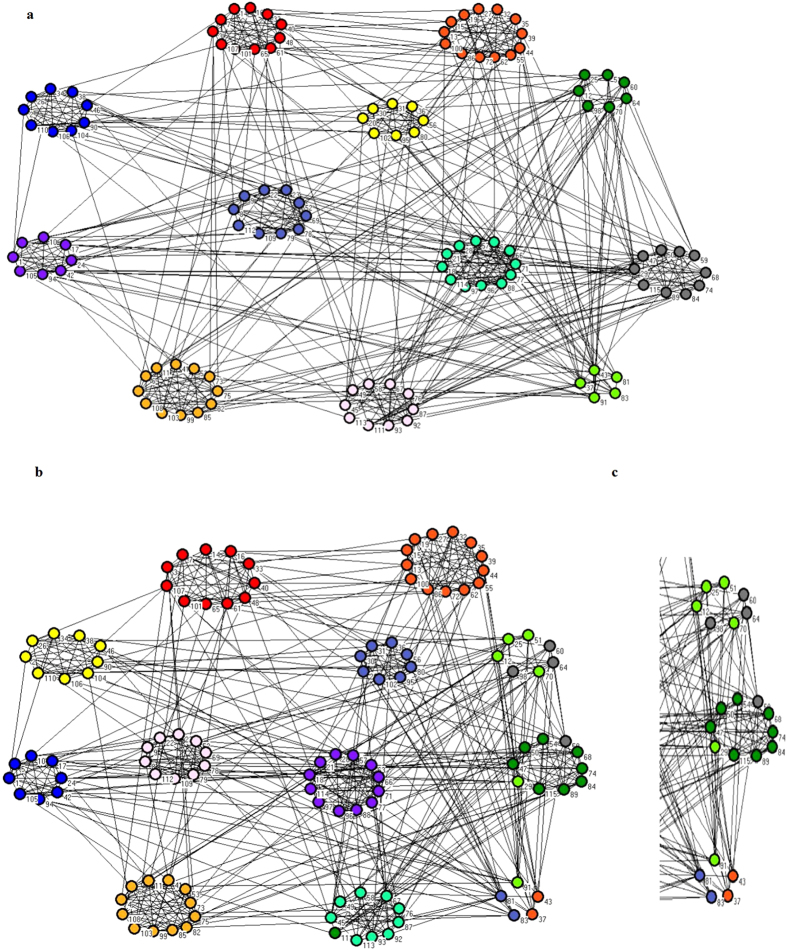
Comparison of three algorithms in time cost.

Table 3 shows that, in terms of the Q value, MOEA/DM’s performance is the same as that for MOPSO, and both (MOEA/DM and MOPSO), in terms of Q value, perform better than MOEA/D. However, as indicated in Table 3 and shown graphically in Fig. 8, in terms of running time, MOEA/DM has the advantage that its running time is substantially less than half that of MOPSO.

### Comparison of algorithms on the football network

#### Football network

When Jantonio Turner^31^ wanted to find more football highlights and discovered that no other all-football channel existed, he founded the football network in August, 1996. He was first mentored by Sheldon Altfeld, who had launched his own channel and who by then was giving seminars to entrepreneurs who wished to begin their own networks.

**Figure 9:**
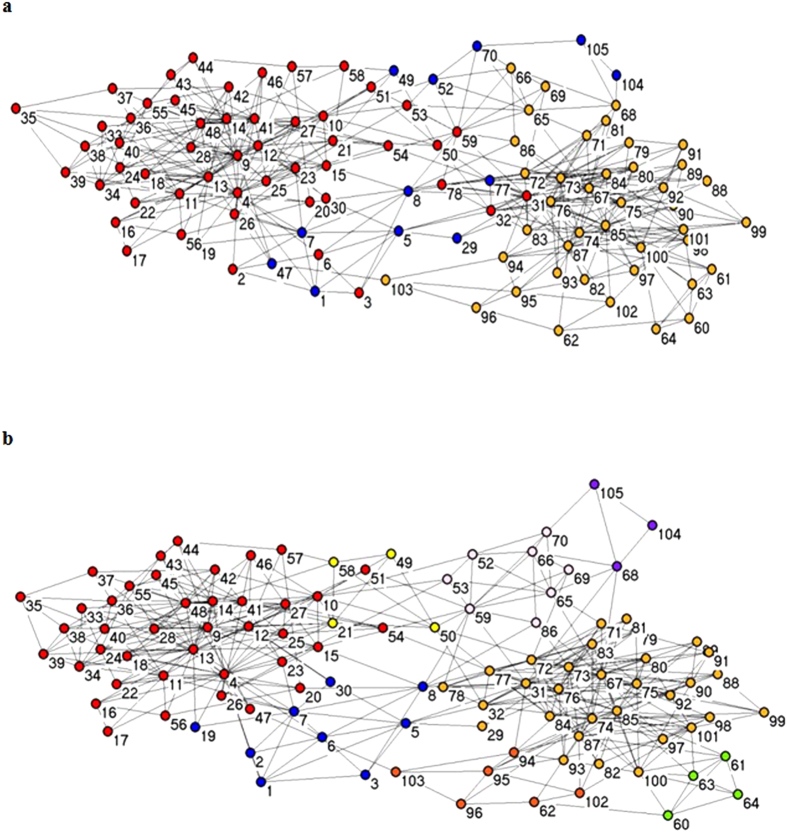
Clustering results on Football club network by MOEA/DM. (**a**) The real structure of Football network. (**b**) The prediction structure of Football network detected by MOEA/DM. (**c**) The apart of (**b**).

The network is divided into twelve categories as shown in Fig. 9(a). Figure 9(b) shows the classification results after using the MOEA/DM algorithm. A comparison of Fig. 9(a) with Fig. 9(b) shows that the football network has a more complex structure than the Dolphin and Karate networks. In the football network, nodes belong to the same classare relatively decentralized. The real network structure, shown in Fig. 9(a), and our predicted network structure, shown in Fig. 9(b), have the same number of categories. From Fig. 9(b) we extracted the three categories on the right and placed them in Fig. 9(c). The three categories in Fig. 9(c) appear to classify the wrong point. The point that marked 58, 29 and the 43, 37, 91 be divided into the wrong position. An analysis of these points reveals that a characteristic they have in common is connecting to other classes is more prevalent than connecting to the edges of their own classes. The MOPSO algorithm divides the network into a like category, but more than 10 points are incorrectly placed.

Table 3 shows that, in terms of the Q value, MOEA/DM’s performance is better than MOEA/D and MOPSO. As indicated in Table 3 and shown graphically in Fig. 8, in trems of running time, MOEA/DM has the advantage that its running time is substantially less than half that of MOPSO.

### Comparison of algorithm on the polbooks network

#### American political book network

The American political book network, based on American political books, is a network of V. Krebs, which has been established on Amazon’s online bookstore. Network edges represent that more readers bought two books simultaneously. This information is obtained from the purchase of books on the web page provided by the “purchase of the book’s readers also buy books.” At the same time, according to the point of view and evaluation of the readers of the Amazon books, Mark Newman divided the node types into three categories: “free,” “conservative,” and “centrist.”

**Figure 10:**
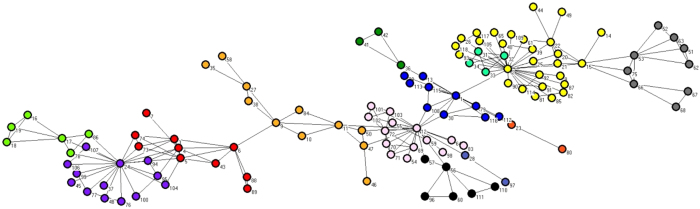
Clustering results on Polbooks club network by MOEA/DM. (**a**) The real structure of American political book network. (**b**) The structure of American political book network detected by MOEA/DM.

The network is divided into three categories as shown in Fig. 10(a). Figure 10(b) shows the classification results after using the MOEA/DM algorithm. A comparison of Fig. 10(a) with Fig. 10(b) shows that the political network has a more complex structure than the Football networks. In the political network, nodes belong to the same classare relatively decentralized. MOEA/DM divides the network into eight category and the part that the color mark red, have divides 9 points are incorrectly placed. The category that color marks blue, have divides four major sub category the color mark orange, green, pink and yellow.

Table 3 shows that, in terms of the Q value, MOEA/DM’s performance is the same as that for MOEA/D, and both (MOEA/DM and MOEA/D), in terms of Q value, perform better than MOPSO. However, as indicated in Table 3 and shown graphically in Fig. 8, in terms of running time, MOEA/DM has the advantage that its running time is substantially less than MOEA/D.

### Experimental results on unknown networks

**Figure 11:**
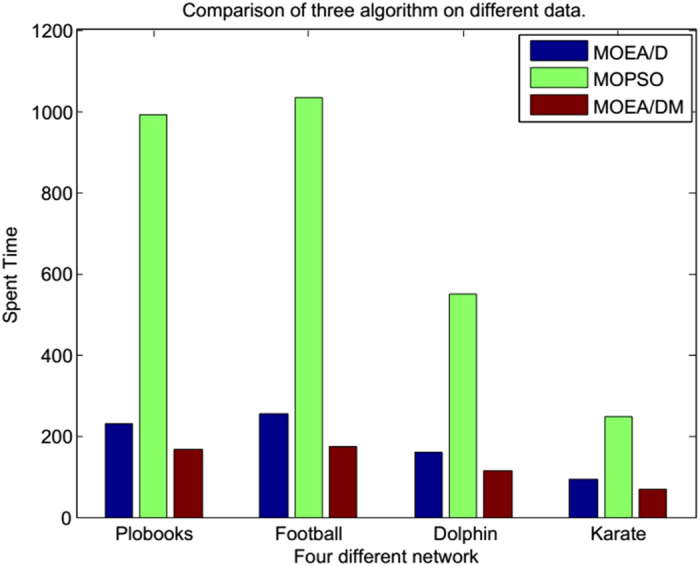
Prediction structure of SFI detected by MOEA/DM.

The SFI^34^ network represents 271 scientists in residence at the Santa Fe Institute, Santa Fe, NM, USA, during any part of calendar year 1999 or 2000, and their collaborators. An edge is drawn between a pair of scientists if they coauthored one or more articles during the same time period. The biggest component of the SFI graph consists of 118 vertices and we only do experiments on this part. Figure 11 show the result of the MOEA/DM. From the picture, the network be divided into twelve category and MOPSO divided into eight category. From the Table 4, the Q value result from MOEA/DM better than the result from MOPSO and the time cost less than MOPSO.

Figure 11 illustrates the results from the algorithm, MOEA/DM, and compare with the MOPSO. In Fig. 11, it very clearly that MOEA/DM splits the network into eight communities, with the same network the algorithm that MOPSO splits network into eight. From the top of Fig. 11 to the bottom, category at the top represents a group of scientists using agent-based models to study problems in economics and traffic flow we shows with the color blue. The second category represents a group of scientists working on mathematical models in ecology we shows with the color red. The third category which made up of four parts, the color in the picture is red, yellow, green, blue, represents a group of scientists working primarily in statistical physics. The two algorithm subdivide this group into four small ones. At the bottom of the figure is a group working primarily on the structure of RNA.

## Concluding remarks

This study introduced an algorithm that combines membrane structure and an evolutionary algorithm, MOEA/DM. In the process of studying the MOEA/D algorithm, it is found that a non-dominated solution corresponds to multiple sub problems. MOEA/DM algorithm, mainly in the number of sub problems and the corresponding solution of each sub problem, improves the number of solutions of one sub problem by trying to reduce the number of sub problems and the addition of film structure to try to ensure that each sub problem has a different number of solutions. Through experiments in the real network, it is found that this improvement has a certain effect. The following is a summary of the three improvements of MOEA/DM:

1. The diversity performance of the proposed algorithm is high because it places the target space on the average weight vector, and the membrane structure is divided into several parts.
2. The time efficiency of the proposed algorithm is higher than those of MOEA/D and MOPSO because the average algorithm to target by the proposed algorithm is divided into several parts: a few particles within the membrane of the evolutionary algorithm.
3. The effect of the proposed algorithm is better than those of MOEA/D and MOPSO, and spends much less time.

Figure 8 illustrates that MOEA/DM has a great advantage in running time. Nevertheless, the results of the experiment in a real network indicate that although MOEA/DM rapidly and accurately locates the real community, it inevitably produces errors in terms of the community number. If a corresponding estimate of the network category is obtained, then the effect is better. In addition to the category of problems, we also determined that certain points (known as the concepts of point classification) still present a high probability of error. Such results are usually generated in the connection between two communities with the same side. If the two goals can be optimized, better results may be obtained.

It is anticipated that the proposed algorithm for complex network clustering can be applied to the field of bioinformatics, such as Disease Gene network^35^,^36^,^37^, DNA binding protein network identification^38^,^39^, protein remote homology detection^40^, etc. It is of interests to consider machine learning methods^41^,^42^,^43^,^44^,^45^ for network clustering. Recently, spiking neural network models, see e.g. refs 46, 47, 48 particularly the ones with-self organizing^49^,^50^ have been a hot topic in the field of machine learning, it is expected to obtain interesting result with this new powerful model.

## Additional Information

**How to cite this article**: Ju, Y. *et al*. Complex Network Clustering by a Multi-objective Evolutionary Algorithm Based on Decomposition and Membrane Structure. *Sci. Rep.* **6**, 33870; doi: 10.1038/srep33870 (2016).

## Figures and Tables

**Figure 1:**
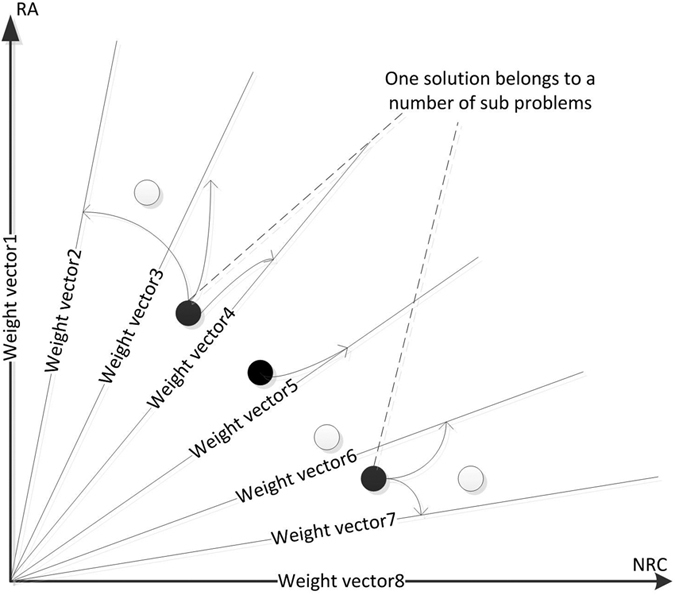
Solution structure of MOEA/D algorithm.

**Figure 2:**
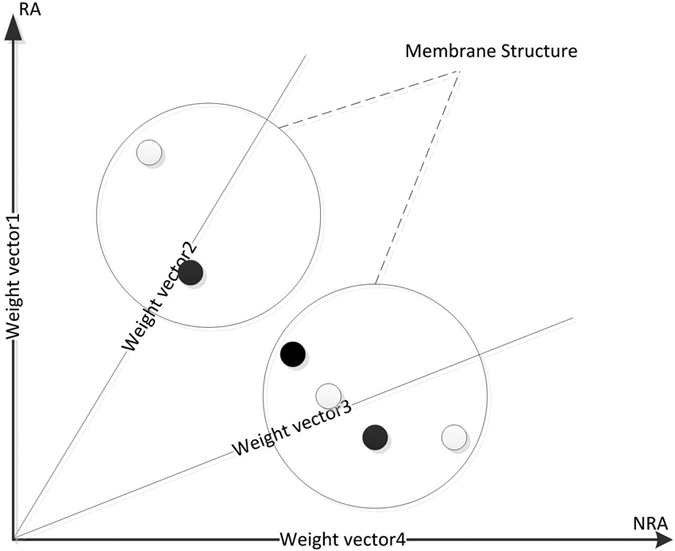
Solution structure of MOEA/DM algorithm.

**Table 1:** Experimental parameters.

Algorithm	Parameters				
	Popsize	CellNum	Niche	PC	PM
MOEA/DM	120	40	13	1	0.06
MOPSO	100	NULL	40	1	0.06
MOEA/D	100	NULL	40	1	0.06

**Table 2:** Network Properties.

Network	Parameters			
	Vertex	Edge	Real	Clusters
Karate	34	78	2	3
Dolphin	62	159	2	3
Football	115	613	12	12
Polbooks	105	882	3	8
SFI	118	200	unknown	13
Netscience	1589	2742	unknown	35

**Table 3:** A comparison of the data on the classification.

Network	MOEA/D		MOPSO		MOEA/DM	
	Q	Time(s)	Q	Time(s)	Q	Time(s)
Karate	0.4198	91.444	0.4198	216.901	0.4198	67.314
Dolphin	0.521	160.596	0.5265	548.290	0.5265	112.845
Football	0.6044	253.406	0.6033	1035.080	0.6046	174.158
Polbooks	0.5269	231.089	0.3600	990.917	0.5269	165.830

**Table 4:** Comparison of the data on the unknown.

Network	MOPSO		MOEA/DM	
	Q	Time(s)	Q	Time(s)
SFI	0.7484	1683.24	0.7486	197.2360
Netscience	0.9503	26790.3	0.9518	578.2089
